# Optimizing endpoints in early phase clinical trials of acute respiratory distress syndrome

**DOI:** 10.1093/ajrccm/aamag238

**Published:** 2026-05-12

**Authors:** Andrew J Boyle, Richard A Greendyk, Bram Rochwerg, Daniel F McAuley, Ewan C Goligher

**Affiliations:** Wellcome-Wolfson Institute for Experimental Medicine, Queen’s University Belfast, Belfast, United Kingdom; Regional Intensive Care Unit, Royal Victoria Hospital, Belfast, United Kingdom; Division of Respirology, Department of Medicine, University Health Network, University of Toronto, Toronto, ON, Canada; Toronto General Hospital Research Institute, Toronto, ON, Canada; Division of Pulmonary, Allergy, and Critical Care Medicine, Columbia University Irving Medical Center, New York, NY, United States; Division of Critical Care, Department of Medicine, McMaster University, Hamilton, ON, Canada; Department of Health Research Methods, Evidence, and Impact, McMaster University, Hamilton, ON, Canada; Wellcome-Wolfson Institute for Experimental Medicine, Queen’s University Belfast, Belfast, United Kingdom; Regional Intensive Care Unit, Royal Victoria Hospital, Belfast, United Kingdom; Division of Respirology, Department of Medicine, University Health Network, University of Toronto, Toronto, ON, Canada; Toronto General Hospital Research Institute, Toronto, ON, Canada; Interdepartmental Division of Critical Care Medicine, University of Toronto, Toronto, ON, Canada

## Introduction

Despite decades of research, there is as yet no proven pharmacotherapy for patients with acute respiratory distress syndrome (ARDS). Nonpharmacological interventions with demonstrated effectiveness in this population are limited to lung-protective ventilation strategies^1^^,^^2^ and venovenous extracorporeal membrane oxygenation.^3^^,^^4^

There are many reasons why clinical trials fail to show a beneficial treatment effect. As our understanding of the biological mechanisms underpinning ARDS have improved,^5^ it has become clear that a basic challenge to demonstrating benefit in clinical trials is that treatment effects may vary between patients according to different biological and physiological phenotypes leading to an attenuated or null average treatment effect.^6-8^ The average treatment effect is further attenuated by risk factors for death unrelated to the targeted mechanism of injury in ARDS, such as comorbidity, frailty, and decisions around withdrawal of organ support.^9^ Intervention-specific factors such as drug dose^10^ and bioactivity^11^ may also be responsible for lack of benefit.

There are multiple opportunities to improve clinical trial design in this field. In this Perspective, we argue that the likelihood of successfully translating preclinical findings to detect signals of efficacy in phase 2 clinical trials in patients with ARDS could be enhanced by using new endpoints with optimal statistical and scientific properties. We outline some of the challenges with current phase 2 trial endpoints, consider what features constitute a suitable endpoint for use in phase 2 clinical trials designed to efficiently identify new therapies in ARDS, and explore the potential for a longitudinal endpoint to improve early-phase clinical trial design and impact.

## Limitations of current endpoints

The strengths and limitations of commonly used endpoints in critical care clinical trials have recently been reviewed in *American Journal of Respiratory and Critical Care Medicine.*^12^ Endpoint selection in phase 2 trials of patients with ARDS is challenging, and we wish to highlight some additional limitations of endpoints commonly used in these settings (Table 1).

**Table 1:** Comparing longitudinal and hierarchical ordinal outcome scales.

	Longitudinal ordinal outcome scale	Hierarchical ordinal outcome scale
**Example**	Glasgow Outcome Scale–Extended (GOS-E)	Organ support–free days
**Strengths**	Can provide longitudinal data, enabling assessment of patient trajectory Can distinguish patterns that share identical organ support–free days totals Statistically more efficient than hierarchical or traditional endpoints (eg, mortality) Can incorporate competing events (eg, death, discharge) Useful for dose-finding studies	Assigns death the worst possible value, without equating death to prolonged duration of therapy Incorporates duration of morbidity Yields a single primary estimand that can be aligned with patient priorities Statistically more efficient than traditional endpoints (eg, mortality) May correlate with longer-term mortality^13^
**Limitations**	Results not easily interpretable Results reflect change in scale position or duration of state occupancy—may not be intuitive to clinicians Changes between points in scale are treated as statistically equivalent but may not be clinically equivalent Statistical methods underpinning results are complex Model assumptions (Markov, proportional odds) require sensitivity analyses Baseline illness severity not accounted for Challenging to estimate power No validated scale in acute respiratory distress syndrome	Measured at a single timepoint Provides single result (eg, days free of organ support) No information on trajectory Interpretation depends on the ranking rules of the hierarchical model (eg, handling ties or handling reinitiation of organ support) Composition of outcome may vary (ie, inclusion of different organ systems) Baseline illness severity not accounted for Challenging to estimate power

First, some endpoints are difficult to ascertain. For example, recent changes to the definition of ARDS allow patients who are receiving high-flow nasal oxygen to be diagnosed with ARDS.^14^ Some physiological outcome measures (eg, oxygenation index) cannot be measured in a nonintubated patient. Moreover, rigorous training may be required to ensure valid and reproducible measurements, such as when using ultrasound-based measures of muscle mass and function (such as might be used in phase 2 trials of drugs for muscle atrophy).

Second, although mortality is a much easier endpoint to obtain and interpret, it generally requires relatively large sample sizes to be able to detect a treatment effect, and this may not be feasible for a phase 2 efficacy study. Prioritizing outcomes other than mortality in phase 2 trials may improve the likelihood of detecting a treatment effect on mortality in phase 3 trials.

Third, patients with ARDS frequently develop extrapulmonary organ dysfunction,^15^ and assessing short-term outcomes in terms of improved lung function without accounting for extrapulmonary organ dysfunction may have contributed to null results in previous pharmacotherapy clinical trials.^16^

Fourth, endpoints that account for organ dysfunction are often measured at a single timepoint (eg, organ support–free days at day 28) or may be averaged over a specified time period (eg, mean Sequential Organ Failure Assessment score to 28 days). While the use of organ support–free days, as in the PANTHER trial (ISRCTN81435672), seeks to incorporate organ failure and mortality into the same ordinal scale, there remain the limitations of this being measured at a single timepoint (day 28). This limitation also applies to ventilator-free days and the hierarchical composite endpoint “alive and ventilator free,”^17^ although the latter may perform better because it does not place equal value on mortality and ongoing mechanical ventilation at day 28. However, none of these approaches capture fluctuations in patient trajectory and would not necessarily reflect “peak” organ failure.

Fifth, while biological endpoint measures reflecting alveolar injury (eg, receptor for advanced glycation end-product) or systemic inflammation (plasma IL-6) are appealing in that they infer the direct modification of ARDS pathophysiology, the biological response to therapy is not always strongly predictive of clinical outcome in critical illness.^16^^,^^18^ It remains difficult to use biological effects to estimate the magnitude of potential treatment effect on a patient-centered endpoint when designing a phase 3 trial.

## What features make for an ideal endpoint in a phase 2 trial in ARDS?

Given these considerations, we suggest a number of features that would be desirable in an endpoint used in phase 2 clinical trials in ARDS^19^^,^^20^:

1. *Responsive to changes in the pathophysiology of ARDS*: ARDS is a complex and heterogenous syndrome that results from a dysregulated host immune response involving multiple biological pathways.^5^ Therapeutics are usually considered if they offer potential to manipulate the underlying immune response, and an ideal endpoint would be sensitive to changes in the underlying biology caused by the studied intervention.
2. *Integrates the effects of an intervention to capture both potential benefit and harm*: It is plausible that an intervention under investigation can have beneficial and harmful effects, and the balance between these forms an important component of the overall assessment of new therapies.^21^ For example, in patients with ARDS, extracorporeal life support techniques may reduce lung injury but with concomitant risks of bleeding, thrombosis, and vascular injury.^4^^,^^22^ Recent^23^ and ongoing (NCT06703073) clinical trials have included risk-benefit assessments, such as the clinical utility index and all-cause mortality-plus score (combining mortality with adverse events), respectively. These innovations represent efforts to capture the net clinical benefit of an intervention in the phase 2 setting. An ideal endpoint would be capable of integrating benefit and harm such that the treatment effect measured by the surrogate endpoint is predictive of the overall treatment effect on patient-centered endpoints.^24^ Furthermore, an ideal endpoint would inform about the trajectory of benefit and harm over time.^21^
3. *Provides information about illness trajectory over time*: In addition to biological complexity, illness trajectory in patients is highly variable and often does not follow a linear pathway. This means that measurement of an outcome at a single timepoint may not sufficiently account for the temporal changes or trajectory of illness.^25^ An ideal endpoint would capture how an intervention can alter illness trajectory beyond a measurement at a single timepoint.
4. *Can account for competing risks such as death or ICU discharge*: Time-dependent endpoints may not be measurable because of competing events (ie, organ function recovery may never be ascertained in a patient who dies on the ventilator before recovery). This reduces statistical power and risks bias if outcomes are missing in a nonrandom fashion.^26^^,^^27^ An endpoint that can account for competing risks would improve the precision of the results and reduce bias.
5. *Holds favorable statistical properties that maximize statistical power*: Maximizing statistical efficiency is an important consideration in clinical trial design. Binary endpoints are less statistically efficient than ordinal endpoints,^28^ which themselves are less efficient than continuous measurements.^29^^,^^30^ In phase 2 trials with smaller sample sizes, maximizing statistical efficiency is important.

## One proposal for a useful endpoint: use of longitudinal ordinal scales

Ordinal endpoints, including scales or categorical scores, are one solution that offers potential to satisfy many of these desirable features in a clinical trial outcome. A well-designed ordinal endpoint could offer information summarizing the reintubation rate, duration of organ failure and hospital admission, and even the functional recovery of patients beyond hospital discharge. These endpoints can be combined in a single ordinal framework that incorporates competing events (eg, death, discharge) and yields outcome summaries that are interpretable to both patients and caregivers.

**Figure 1:**
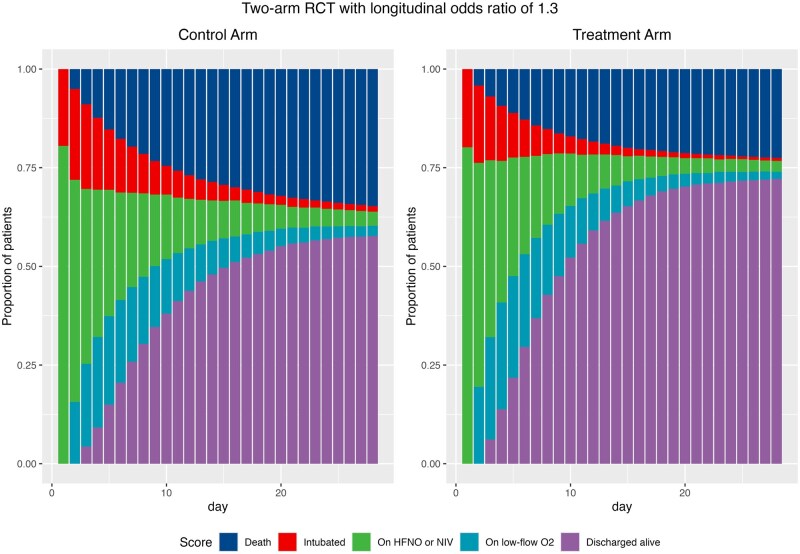
Simulated daily distributions of an ordinal clinical status scale in a 2-arm randomized trial under a proportional-odds treatment effect (OR = 1.3). The 5-point scale includes death, intubated, HFNO/NIV, low-flow oxygen, and discharged alive. Each panel shows the proportion of patients in each state over 28 days in the control and treatment arms. The OR is illustrative; clinical interpretability can be obtained by translating model effects into absolute differences in state occupancy at prespecified time points (eg, day 14 or day 28) and differences in time spent in favorable states, using baseline trajectories representative of acute respiratory distress syndrome populations. Abbreviations: HFNO, High-flow nasal oxygen; NIV, Non-invasive ventilation; O_2_, oxygen; OR, odds ratio; RCT, randomized controlled trial.

Ordinal endpoints can be assessed longitudinally at repeated intervals over time in a clinical trial setting (Figure 1).^31^ Longitudinal endpoints capture information about patient trajectory and can facilitate analysis of time-dependent effects.^32^ In this setting, the estimand of interest is typically a *marginal* treatment effect that summarizes the average trajectory across all randomized patients, rather than conditional effects restricted to survivors or those remaining in hospital at each timepoint. For example, investigators might contrast treatment groups in terms of the mean number of days spent alive and in more favorable categories of respiratory support over the follow-up period or in the rate of improvement across levels of the scale over time.

Additionally, the use of repeated measures can increase statistical information and can enhance power to detect such treatment effects. Simulation studies of longitudinal ordinal outcomes in critical care suggest that trajectory-based endpoints can improve statistical efficiency, particularly when interventions influence the rate or timing of improvement rather than only affecting a patient’s status at the end of the follow-up period.^13^ Other reanalyses of critical care trials using multistate frameworks demonstrate how modeling patient trajectories can clarify treatment effects by capturing differences in timing, transitions, and competing events that are not apparent from summary endpoints at a single timepoint.^33^ Furthermore, even when the treatment effect at a particular point in time is of primary concern, longitudinal analyses can provide advantages over traditional cross-sectional analyses by estimating the intervention effect at the end of the study period with greater precision.^34^^,^^35^

Beyond their role as efficacy endpoints, longitudinal ordinal endpoints may also be useful in dose-finding early-phase trials. In oncology, longitudinal ordinal models of graded toxicities have been used to inform dose-escalation decisions, allowing more efficient use of repeated safety data while avoiding dichotomization of adverse event severity.^36^ Such approaches have performed well when compared with traditional dose-finding frameworks and have been successfully applied across multiple phase 1 and 2 studies.^37^ These methods may be most relevant for phase 2 trials in ARDS, where dose optimization would ideally balance evolving safety signals with the longitudinal clinical response to an intervention.

### Analytical considerations

Bayesian ordinal transition models offer one flexible framework for estimating these marginal, trajectory-based estimands from longitudinal ordinal scales.^38^ In contrast to composite summary outcomes such as ventilator-free days, which collapse death, prolonged ventilation, and recovery into a single scalar measure at a fixed timepoint (even if they are analyzed as an ordinal scale), explicitly state-based ordinal clinical status scales preserve these elements as distinct states throughout the duration of follow-up. As a result, treatment effects reflect differences in the distribution of clinical states over time and are estimated marginally across all randomized patients. Transition-based modeling frameworks, such as Bayesian ordinal transition models, allow death and recovery to be represented as distinct terminal states, enabling estimation of clinically interpretable quantities such as state occupancy probabilities and mean days spent in favorable states.^38^ This approach captures the full clinical trajectory and avoids the challenged posed by composite endpoints where distinct failure modes (eg, death vs prolonged ventilation) yield identical endpoint values. More broadly, Bayesian analyses of longitudinal outcomes may support phase 2 decision-making by enabling estimation of predictive probabilities of success in subsequent phase 3 trials, as has been demonstrated in adaptive oncology platforms such as the I-SPY 2 Trial.^39^ In ARDS, where disease trajectories are short and validated surrogate endpoints are lacking, longitudinal ordinal outcomes measured on the causal pathway of recovery may provide a pragmatic basis for such predictions without reliance on external surrogates.

Evidence from other disease areas also suggests that the performance of longitudinal ordinal endpoints may depend on the severity of illness in the enrolled population. In a simulation study comparing multiple endpoint types for an influenza trial, the investigators found that longitudinal ordinal models were generally less powerful than single-timepoint ordinal analyses, except in scenarios that enrolled more severely ill patients with prolonged hospitalization, conditions more analogous to ARDS populations.^30^ In those settings, the longitudinal ordinal approach performed best, perhaps because it better captured extended trajectories of organ support and recovery. This highlights that while longitudinal ordinal endpoints are not necessarily universally superior to other endpoints, they may offer advantages in populations such as ARDS, where clinical trajectories are prolonged and characterized by sustained organ dysfunction.

### Use of longitudinal ordinal outcomes in clinical trials

While no longitudinal endpoint has been specifically validated for ARDS, longitudinal ordinal severity scales have been used in other randomized controlled trials (RCTs).^25^^,^^40-43^ These may serve as models for exploring the integration of longitudinal ordinal endpoint assessments into clinical trials of ARDS.

For example, the Glasgow Outcome Scale–Extended (GOS-E), which grades functional status from a score of 1 (death) to 8 (upper good recovery) (Table 2), is frequently assessed longitudinally to evaluate the trajectory of recovery and evaluate the time-dependent effects of interventions focused on rehabilitation after traumatic brain injury (TBI).^32^ GOS-E has successfully been used to track recovery over time in several TBI clinical trials^50-52^ with demonstrated improvements in statistical efficiency.^28^

**Table 2:** Examples of longitudinal ordinal endpoints.

Ordinal scale name	Levels of the scale	Assessment frequency	Follow-up duration	Primary analytical approach
**DOSE**	Dead Receiving ECMO Ventilated but not receiving ECMO In an ICU but not ventilated In a hospital ward Discharged from the hospital	Daily	90 days	Longitudinal Markov ordinal model^13^
**GOS-E**	Death Vegetative state Lower severe disability Upper severe disability Lower moderate disability Upper moderate disability Lower good recovery Upper good recovery	3 months, 6 months, 12 months	12 months	Varies; approaches include binary (dichotomized) logistic regression, sliding dichotomy method, and ordinal logistic regression^44^ ^,^ ^45^
**SepTiC Clinical State Scale**	In-hospital with organ support In-hospital without organ support Discharged from hospital	Daily	90 days	Markov transition model^46^ ^,^ ^47^
**WHO-CPS**	Uninfected; no viral RNA detected Asymptomatic; viral RNA detected Symptomatic; independent Symptomatic; assistance needed Hospitalized; no oxygen therapy Hospitalized; oxygen by mask or nasal prongs Hospitalized; oxygen by NIV or high-flow Intubation and mechanical ventilation, pO₂/FiO₂ ≥150 or SpO₂/FiO₂ ≥200 Mechanical ventilation pO₂/FiO₂ <150 (SpO₂/FiO₂ <200) or vasopressors Mechanical ventilation pO₂/FiO₂ <150 and vasopressors, dialysis, or ECMO Dead	Daily	Up to 30 days	Varies; approaches include ordinal logistic regression (proportional odds model), time-to-event analysis, cross-sectional ordinal analysis with proportional odds model, Bayesian ordinal transition model^38^ ^,^ ^43^ ^,^ ^48^ ^,^ ^49^

Abbreviations: DOSE, daily organ support for patients on extracorporeal membrane oxygenation outcome; ECMO, extracorporeal membrane oxygenation; FiO_2_, inspired fraction of oxygen; GOS-E, Glasgow Outcome Scale–Extended; ICU, intensive care unit; NIV, non-invasive ventilation; pO_2_, partial pressure of oxygen; SepTiC, Sepsis trials in Critical Care; SpO_2_, oxygen saturation; WHO-CPS, World Health Organization COVID-19 Clinical Progression Scale.

The ongoing SepTiC trial provides another example of the novel use of longitudinal ordinal endpoints in critical care research (ISRCTN80791572). The trial, which is evaluating fluid management strategies and therapeutics for patients with sepsis, uses a primary outcome that combines mortality at 90 days with clinical state as an ordinal scale (in-hospital with organ support, in-hospital without organ support, discharged from hospital) with repeated observations over time (Table 2).

In the context of respiratory failure, the recently described “daily organ support for patients on extracorporeal membrane oxygenation” (DOSE) ordinal endpoint demonstrated substantially greater statistical efficiency than 28-day mortality in simulation studies (Table 2). Using assumptions derived from an observational prospective registry, the investigators found that the longitudinal DOSE model offered up to 56% more power than a binary 28-day mortality endpoint in a hypothetical 2-arm trial with 400 patients per group and an odds ratio of 1.2.^13^ However, these power estimates depend on simulation choices (such as transition structures, effect-size assumptions, and correlation patterns), which were not fully detailed in the publication. As such, the magnitude of the reported efficiency gain should be interpreted cautiously and may differ in patients with ARDS or other critically ill populations with different illness trajectories or treatment effect profiles. Nevertheless, the DOSE outcome illustrates the potential for longitudinal ordinal endpoints to improve statistical efficiency by capturing more of the patient’s clinical trajectory over time.

Early in the COVID-19 pandemic, a minimum set of common endpoint measures for studies enrolling patients with COVID-19 was established.^43^ This framework included a 10-point ordinal clinical progression scale, with categories based on functional status and level of oxygen support (Table 2). A modified version of this scale was used in a clinical trial evaluating remdesivir for the treatment of COVID-19.^48^ The ordinal scale was assessed longitudinally and incorporated into the primary outcome, time to recovery (defined as meeting criteria for category 1, 2, or 3 on the ordinal scale), as well as secondary outcomes including ordinal scale at multiple endpoints and mean change in ordinal scale between multiple timepoints. While there was no difference in mortality between those receiving remdesivir versus placebo, investigators detected a benefit with remdesivir in these longitudinal ordinal scale–informed primary and secondary outcomes.

## Limitations of longitudinal ordinal endpoints

Longitudinal ordinal endpoints hold promise for capturing nuanced treatment effects in early-phase ARDS clinical trials, but several important limitations must be addressed.

First, these endpoints rely on complex statistical methods that require careful validation before widespread adoption. In a scoping review of RCTs using ordinal endpoints, 60% of trials did not assess or report the robustness of the assumptions underpinning the analysis of the ordinal endpoint, highlighting the need for more transparent reporting and clearer guidance if these endpoints are to be implemented more broadly.^53^ Although violations of the proportional odds or first-order Markov assumptions may occur in longitudinal models, several methodological approaches are available to assess and mitigate their impact. For ordinal analyses, proportional odds violations can be addressed using partial proportional odds models or alternative link functions. Furthermore, recent work by Harrell suggests that violations of the proportional odds assumption may not actually prevent the model from providing a reasonable treatment effect assessment.^54^ For transition-based models, recent methodological ­guidance recommends flexible multistate or semi-Markov specifications, which allow transition rates to depend on time spent in a state or on time-varying hazards rather than assuming a strictly Markov process. Sensitivity analyses using alternative transition structures can further evaluate the robustness of key inferences.^33^

Second, powering trials with longitudinal ordinal endpoints can be challenging. Ordinal endpoints often yield multiple interrelated measures rather than a single endpoint, creating uncertainty about what, specifically, is being powered. Prespecified models may fail to converge if patient trajectories differ from expectations, and there are currently no widely accepted standards for presenting or interpreting results. Violations of key assumptions—such as the Markov property, which assumes transitions depend only on the current state—can introduce bias, which may be particularly relevant to critically ill patient populations in whom prior states often influence subsequent outcomes. In addition, repeated measures may amplify postrandomization imbalances if not properly addressed.^12^

Third, interpretation of longitudinal ordinal endpoints can be challenging. Longitudinal odds ratios represent the odds of being in a higher (or lower) category, which may not be intuitively meaningful for many clinicians. Varying treatment effects across endpoint categories also require robust modeling, yet such complexity can limit translation of results into practice.^55^ Furthermore, one-unit changes between points on an ordinal scale may not be of equivalent importance For example, moving from mechanical ventilation to high-flow oxygen may be more clinically meaningful than moving from high-flow to standard oxygen, despite both being a single-category change (Figure 1).^56^

Fourth, longitudinal ordinal endpoints may reveal differences in early clinical trajectories even when both treatment groups ultimately converge to similar end states, such as similar mortality or recovery rates by day 28. However, in some settings these trajectory effects may still be clinically meaningful even if there is no difference in the final outcome. Thus, the finding of convergence in an early phase trial utilizing a longitudinal endpoint should be carefully weighed in the decision to proceed to phase 3.

## Conclusions

Clinical trials in ARDS have used a broad range of endpoint measures, including biological, clinical, and physiological endpoints—all with inherent limitations, including poor correlation with commonly used phase 3 clinical trial endpoints, lack of patient-centeredness, inability to inform expected treatment effect sizes, and unfavorable statistical properties related to power and sample size. Longitudinal ordinal endpoints have potential to enhance the efficiency of phase 2 clinical trials in ARDS, and this may substantially increase our chances of finding effective pharmacotherapies for these patients pending regulatory guidance on their use.

## Supplementary Material

aamag238_Supplementary_Data
